# Recognizing RNA structural motifs in HT-SELEX data for ribosomal protein S15

**DOI:** 10.1186/s12859-017-1704-y

**Published:** 2017-06-06

**Authors:** Shermin Pei, Betty L. Slinger, Michelle M. Meyer

**Affiliations:** Boston College, 140 Commonwealth Ave., 02467 Chestnut Hill, USA

**Keywords:** SELEX, Ribosomal protein, Motif, S15

## Abstract

**Background:**

Proteins recognize many different aspects of RNA ranging from single stranded regions to discrete secondary or tertiary structures. High-throughput sequencing (HTS) of in vitro selected populations offers a large scale method to study RNA-proteins interactions. However, most existing analysis methods require that the binding motifs are enriched in the population relative to earlier rounds, and that motifs are found in a loop or single stranded region of the potential RNA secondary structure. Such methods do not generalize to all RNA-protein interaction as some RNA binding proteins specifically recognize more complex structures such as double stranded RNA.

**Results:**

In this study, we use HT-SELEX derived populations to study the landscape of RNAs that interact with *Geobacillus kaustophilus* ribosomal protein S15. Our data show high sequence and structure diversity and proved intractable to existing methods. Conventional programs identified some sequence motifs, but these are found in less than 5-10% of the total sequence pool. Therefore, we developed a novel framework to analyze HT-SELEX data. Our process accounts for both sequence and structure components by abstracting the overall secondary structure into smaller substructures composed of a single base-pair stack, which allows us to leverage existing approaches already used in k-mer analysis to identify enriched motifs. By focusing on secondary structure motifs composed of specific two base-pair stacks, we identified significantly enriched or depleted structure motifs relative to earlier rounds.

**Conclusions:**

Discrete substructures are likely to be important to RNA-protein interactions, but they are difficult to elucidate. Substructures can help make highly diverse sequence data more tractable. The structure motifs provide limited accuracy in predicting enrichment suggesting that *G. kaustophilus* S15 can either recognize many different secondary structure motifs or some aspects of the interaction are not captured by the analysis. This highlights the importance of considering secondary and tertiary structure elements and their role in RNA-protein interactions.

**Electronic supplementary material:**

The online version of this article (doi:10.1186/s12859-017-1704-y) contains supplementary material, which is available to authorized users.

## Background

RNA-binding proteins (RBPs) play essential cellular roles that range from co- and post-transcriptional regulation of mRNA transcripts [1, 2], to stabilization of macromolecular complexes such as the ribosome [3]. In this genomic era, the imperative to utilize primary sequence data to elucidate the relationship between an RBP, its recognition site, and its function, is only growing [4]. Identifying the binding sites for RBPs is an important task toward unraveling gene regulatory networks [5]. However, prediction of RBP interaction sites remains a challenge. Unlike DNA-binding proteins (DBPs), RBPs may recognize features of single-stranded RNA, double-stranded RNA, or even three-dimensional tertiary structures [6]. Therefore, RNA structure must be taken into account in assessment of potential binding-sites. One method of experimentally identifying the constraints on an DBP or RBP recognition site is SELEX (Systematic Evolution of Ligands by Exponential Enrichment) [7, 8]. SELEX is an iterative in vitro selection technique that allows researchers to identify nucleic acids that interact with a target ligand. Analysis of the sequences resulting from a SELEX experiment can be used to confirm the specificity of a binding site, or illuminate how RNA structural plasticity may enable multiple sequences to present a similar three-dimensional motif to the protein [9].

With the advent of next-generation sequencing, high-throughput sequencing-SELEX (HT-SELEX) has become an even more powerful approach to explore RNA-protein interactions. Sequence conservation within the selected population gives insight into important nucleotides, circumventing the need for laborious follow-up experiments to identify key regions of the selected sequences. The nucleotide differences between closely related sequences effectively explore local sequence space [10–13], and highly conserved areas point toward functionally important positions. Using such patterns of variation and conservation, information about the critical sequence motifs responsible for binding is revealed. Furthermore, sequencing intermediate rounds of the selection process allows ancestral sequences to be determined rather than inferred, and sequences that enrich over several SELEX rounds are more likely to be high affinity binders [14]. In addition, due to the high diversity of sequences undergoing selection, multiple possible and distinct binding motifs or structures can be discovered in a single experiment.

One downside of HT-SELEX approaches is the size and complexity of data that may be generated, especially from large randomized nucleotide populations. Typically, the RNA selection process starts with a pool of molecules on the order of 10^12^−10^14^ sequences, which can still be dwarfed by the total number of possible sequences (4^sequence length^). In the ideal circumstance, over the course of a SELEX experiment, the sequence pool will converge on a small number of sequences that reflect a shared potential binding motif. If the entire sequence pool is sequenced, then these features should stand out as prevalent and enriching sequences within the population. In practice, given the size of the populations, under-sampling remains a significant hurdle. Thus, often only a sparse view of the RNA-binding pool is provided [11, 15, 16], potentially obscuring patterns that might be apparent from more thorough analysis.

Typical analysis of HT-SELEX data involves the identification of the RNA-protein binding motif. This analysis is distinct from transcription factor identification in that there can be multiple potential binding motifs and these motifs are likely to have a secondary structure context [17–19]. Programs found in the MEME suite [20] such as MEME, GLAM2 [21], and DREME [22] can be applied to the HTS data to identify binding motifs. MEME and DREME are designed to find contiguous sequence motifs. GLAM2 identifies motifs that can include short-gaps. However, there are a some of drawbacks to using these tools. Due to their algorithmic complexity, MEME and GLAM2 are not equipped to use large magnitudes of sequence data [21, 23]. DREME’s run time scales linearly with the data set size, but this is still not sufficient to keep pace with larger HTS data sets. Additionally, these programs ignore any potential secondary structure, which can hinder their ability to find the putative binding motifs.

To identify sequence-structure motifs, there are programs such as MEMERIS [17], RNAcontext [18, 24], AptaMotif [25], MPBind [26], GraphProt [27], RCK [28], AptaNI [29], and AptaTRACE [30]. MEMERIS specifically identifies motifs found in the loop regions of the secondary structure, but like MEME, it is not designed for HTS data. RNAContext and RCK use sequence and structure information to find RNA binding motifs, but they require a large number of both binder and non-binder motifs in order to determine the motif enrichment because it is assumed that the binding motif is contiguous and is present in majority of binders and not in the non-binders. MPbind uses a k-mer approach to identify contiguous binding motifs by identifying prominent subsequences that are enriched between selection rounds. GraphProt leverages secondary structure to identify binding motifs, but it also requires data on binders and non-binders alike. AptaMotif is designed to analyze low throughput SELEX data, but it has been extended in the form of AptaNI, which restricts the motif search to loop regions of the structure. AptaTRACE is a state-of-the-art HT-SELEX motif identification tool that takes into account both sequence and structure to identify binding motifs. Overall, many of these programs focus on identifying contiguous motifs while using secondary structure to restrict the search to single stranded regions.

HT-SELEX analysis techniques have been successfully applied to identify short sequence motifs responsible for RNA-protein interactions [31, 32], typically located in internal loop regions [33]. While this type of analysis is effective for many RBP binding-motifs, particularly those that involve recognition of single-stranded regions of RNA, not all RBPs conform to such recognition patterns [6]. In many cases an RBP may interact with complex tertiary structure motifs, and in some cases with multiple complex structures. Some RNA binding proteins, such as ADAR or Staufen, specifically recognize double stranded RNA. These binding proteins target a structure containing 12 or 16 base-pairs, such as a single stem or co-axially stacked stems [34, 35].

In *Escherichia coli*, several ribosomal proteins interact not only with the rRNA, but also with structured portions of their own transcripts. These interactions allow stoichiometric production of ribosomal proteins by inhibiting transcription or translation [36]. While in some cases the mRNA structures are apparent mimics of the rRNA-binding sites, in other cases similarity is not obvious [37]. In addition, many of the mRNA structures responsible for this regulation in *E. coli* are narrowly distributed to only a few bacteria [38].

Ribosomal protein S15 is a particularly interesting example of ribosomal protein regulation. S15 is a conserved protein across bacterial phyla, and in some bacteria it is auto-regulated at the translational level [39]. However, species within different bacterial phyla use distinct mRNA structures to accomplish the same regulatory task [38, 40, 41]. There are at least four distinct mRNA secondary structures that regulate in response to S15, each constrained to a single bacterial phyla. Each structure likely evolved independently, thus mRNA interactions with homologous S15 proteins are not necessarily conserved. In contrast, both the S15 protein and its 16S rRNA binding site are highly conserved among different lineages of bacteria. While previous work has identified the critical motifs in the 16S rRNA (a GU/GC within a paired region and a 3-helix junction) responsible for efficient S15 binding in *E. coli* and *Thermus thermophilus*, various mRNA structures can bind S15 despite containing some but not necessarily all of the 16S rRNA binding determinants [42–44]. Furthermore, not all homologous S15 proteins are interchangeable regulators between different bacterial species, indicating some target specificity [45]. Recently, we identified a set of SELEX derived RNA structures that bind *Geobacillus kaustophillus* S15 [46]. The identified RNAs are distinct from known natural regulators, but several still regulate gene expression in response to S15. Just as in nature, a high degree of sequence and structure diversity was found in this study, suggesting that the natural diversity of RNA regulation is not solely due to differences between S15 protein homologs.

In this work, we analyze the intermediate and final rounds of SELEX against *G. kaustophilus* S15 using high-throughput sequencing in order to better understand the diversity of potential RNA structures that interact with S15. The complex nature of the S15-binding site is a likely factor contributing to the high sequence diversity observed in our data. To elucidate any sequence-structure motifs, we developed an analysis approach that simultaneously considers the sequence and structure to identify a discontinuous double-stranded binding motif. By treating RNA structure as a set of discrete substructures, we identify enriched structure elements associated with the RNA-S15 binding site. In particular, we find many potential binding motifs that are significantly enriched over the course of selection. Combining these motifs and experimentally validated binders, we build a model to separate specific and non-specific S15 binders. Overall, we find that S15 heavily relies on the structure for recognition of its target.

## Results

### Characterization of selected population

**Fig. 1:**
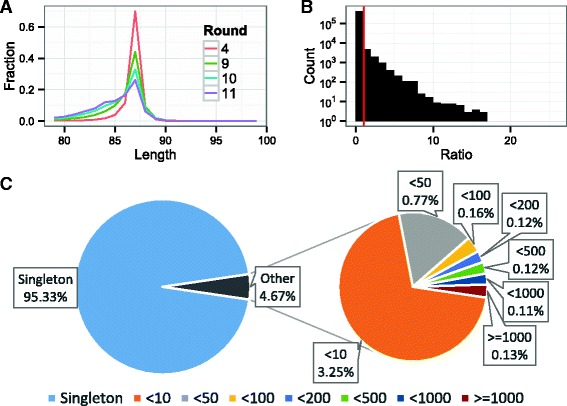
**a** Distribution of read lengths shows most reads are the expected length of 87 nt. **b** Distribution of sequence enrichment of multiton sequences in rounds 10 and 11. The enrichment is normalized to the total number of reads in the round. The red line indicates no enrichment (ratio = 1). **c** Majority of the sequences in our total sequence pool are singleton sequences

We characterized the reads resulting from sequencing reverse transcribed and amplified products of SELEX rounds 4, 9, 10, and 11 by examining read lengths, sequence enrichment, and diversity. There were 32,866,739 total pair-end reads of which 5,584,124 reads were forward strand and passed quality filters (Table 1) (See Methods: High-throughput sequencing). Most of the reads are the expected length of 87 nt (Fig. 1 a). The reads tend to become shorter in rounds 9, 10, and 11 compared to round 4. Additionally, we noticed there was an increase in fragments of approximately 79 nt (Additional file 1: Table S1). These shorter fragments are most likely preferentially amplified during PCR compared to longer fragments. However, such individuals examined using filter-binding assays do not bind S15 specifically. We found that ≈2% of sequences from rounds 10 and 11 were enriched during the SELEX process (Fig. 1 b) indicating the selection is likely enriching for specifically binding sequences. Finally, there was significant sequence diversity in the sequence pool. 95.33% of sequences appeared only once (singleton) and of the sequences that appeared more than once (multiton), 69.5% were seen fewer than 10 times (Fig. 1 c).

**Table 1:** Total number reads by round before and after filtering

Round	Unfiltered	Filtered
4	10,978,044	4,150,081
9	10,854,647	407,138
10	5,764,497	481,763
11	5,269,551	545,142
Total	32,866,739	5,584,124

### Identification of global similarity between clusters

Despite the large number of singleton sequences present in our data, there may be a large number of similar or related sequences (similar primary or secondary structure) present. Therefore, to reduce the complexity of our data and identify related sequences, we grouped sequences with high sequence identity together. Due to the number of sequences, identification of common sequence or structure using pairwise comparisons is computationally prohibitive. There are several readily available programs that cluster based on sequence, such as CD-HIT [47], or cluster based on sequence and structure, such as RNAclust.pl + LocARNA [48]. However, most structure clustering tools are not applicable to the HTS data. RNAclust.pl is designed to cluster < 1000 sequences and LocARNA (and its derivatives LocARNA-P [49] and SPARSE [50]) are designed to simultaneously use sequence and structure to create multiple sequence alignments from homologous sequences, not the large and diverse set of sequences we obtained through SELEX. While CD-HIT only compares sequences, similar sequences are likely to fold into similar structure. Therefore, we used CD-HIT, a fast and widely-used program for nucleic acid clustering that utilizes heuristics to significantly reduce run time.

**Fig. 2:**
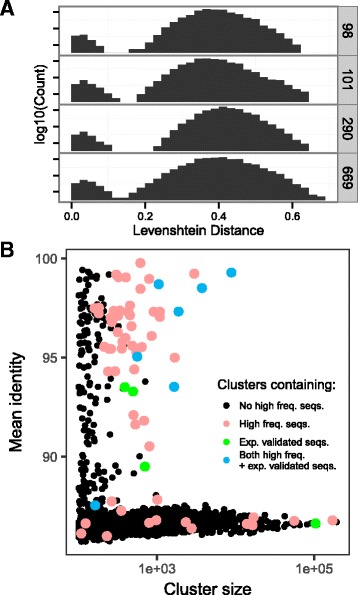
**a** Histogram of normalized Levenshtein distance from the top 4 high frequency sequences (Seq. ID: 98, 101, 290, 669) shows a clear cluster cutoff at distance 10%. Within the cluster, there is a decrease in the frequency of sequences further from the center indicating sequence clusters containing high frequency sequences are valid. **b** Plot of the CD-HIT clustering data represented as cluster size vs mean percent identity to cluster seed (diffuseness). In *red* are the clusters containing high frequency sequences with more than 100 read counts. In *blue* are clusters containing high frequency sequences with more than 100 read counts, which have been experimentally examined for binding to S15 (Table 6). In *green* are sequences experimentally tested that are from the clusters that do not contain high frequency sequences

We established a clustering threshold by examining sequence similarity to high frequency sequences. Examining the distribution of sequence distance around high frequency sequences shows a clear separation at 10% normalized edit distance, which is equivalent to 90% sequence identity (Fig. 2 a). Clusters formed around the most frequent sequences are distinct, as seen by having lower within-cluster distance than between-cluster distance. This trend continues to be true for all high frequency sequences (Additional file 1: Figure S1). Because CD-HIT run time increases proportionally to the number of clusters (which increases dramatically as the sequence identify threshold is increased), we reduced the run time by using an 85% sequence identity as the clustering threshold.

**Fig. 3:**
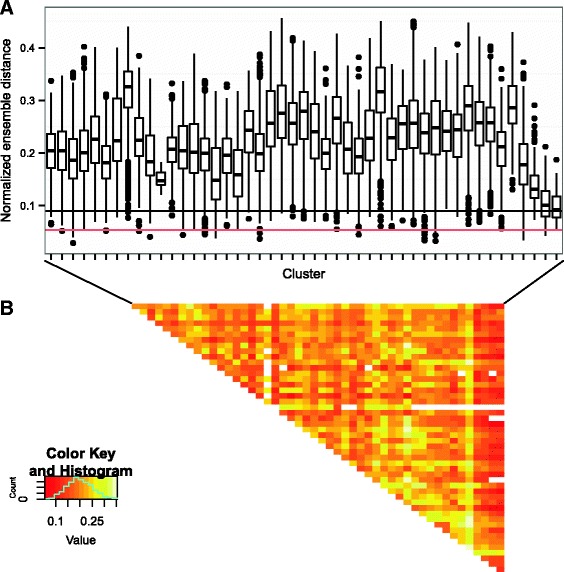
**a** Distribution of inter-cluster ensemble distances from cluster 6062, which contains the most frequent sequence. Clusters selected for comparison included clusters with >100 distinct sequences, >90% mean identity to the seed. To get a distance distribution when comparing clusters to cluster 6062, individual sequences of the same length from the given cluster and cluster 6062 were compared in an all-against-all fashion. As a reference, the median intra-cluster distance for cluster 6062 was 0.0898 (black line) and the first-quartile was 0.0536 (red line). **b** Representing all selected cluster pair-wise comparisons distance distributions in a heatmap shows that on average, clusters differ from other clusters by 0.2. In general, many of the structures are distinct from those of other cluster structures

Given the observed sequence diversity across our clusters, we also assessed whether any similar global secondary structures were shared between clusters. Clustering similar sequences together reduces the number of structure prediction operations because a representative cluster structure can be quickly determined by sampling and folding a small number of sequences (See Methods: Intra/inter-cluster ensemble distance). Therefore for these comparisons, we focus on clusters with > 90% similarity (Fig.2 b). Using this method, we find that sequence clusters are also effective structure clusters because the intra-cluster structure distance (median distance of 0.0898, Additional file 1: Figure S2) is lower compared to the inter-cluster structure distance (Fig. 3). Additionally, pairwise comparisons of the clusters shows higher inter-cluster structure distance, indicating there is no globally similar structure shared between clusters. While some clusters appear to have similar structure (Fig. 3 b), upon closer inspection, this similarity is an artifact caused by comparing a limited number of structures from each cluster (See Additional file 1: Methods, Table S2, Figure S3).

### Identification of sequence motifs

The high cluster count made it difficult to extract meaningful sequence or structure patterns in the data. In order to identify any common short sequence motifs, we started with sequence based approaches for motif identification because there are a variety of existing tools (summarized in Table 2). Many tools for motif identification are found in the MEME suite (MEME, GLAM2, DREME). In particular, MEME and GLAM2 are not designed to process HTS data. To overcome the large number of sequences in our dataset and differences in the number of sequences in each round, we repeatedly sampled 10^5^ sequences from each round for a total of 4∗10^5^ sequences. This sample size represents approximately 20% of rounds 9, 10, and 11, but only 2.5% of round 4. Such sampling allows us to compare a number of different methodologies for analyzing the data, regardless of whether they are explicitly designed for large data sets. However, while MEME is powerful and can identify transcription factor binding sites, in practice the algorithmic complexity limits the data to < 1000 sequences [23]. GLAM2 is able to identify gapped motifs and tolerates larger data sets, but it does not find any significant motifs (E-value = 1) in our data (Additional file 1: Figure S4). We also applied DREME to find short k-mers (3≤*k*≤8), and some of the top motifs with more than 10^4^ occurrences are significant (Table 3). These motifs are repeatedly found in multiple resamplings of the data; however, they are only found in 1.2-5% of the total sequence pool.

**Table 2:** Comparison to existing tools

Software	Run Time
MEME	N/A
DREME	≈3 hrs
GLAM2	≈1 week
AptaTrace	≈5 hrs
AptaTrace (33% sampling)	≈21 hrs
AptaTrace (full data)	≈70 hrs
RNAcontext/RCK	≈1 week
NCM	≈10 hrs

Unless noted otherwise, all software were run using a sample size of 4∗10^5^ sequences

**Table 3:** Top DREME motifs with > 10^4^ observations

Motif	E-Value	Percent sequences containing motif(%)
YACTGCT	2.4e-2784	1.2
WTAYGGA	5.6e-1525	1.5
WCCRAG	1.3e-515	5.0

Where R = A or G; Y = C or T; W = A or T

Additionally, we applied other state of the art programs for identifying binding motifs in HT-SELEX data (Table 2). AptaTRACE returned no significant results with our sampled data (10^5^ sequences per round). Upon increasing the sample size to 33% of each round, AptaTRACE returned a set of significant motifs, the top five of which are shown on Table 4 (full results shown in Additional file 1: Figure S5). Notably the top motif is similar to the top motif identified by DREME (ACTGCT). However, all the seed sequences are present at < 10% of the final population (seed frequency), and even partially degenerate motif sequences typically represent < 15% of the population (Additional file 1: Figure S5). Resampling the data did not substantially alter the top motifs identified or the frequencies with which they appeared in the data. We subsequently ran AptaTRACE on our entire data set and obtained a slightly different set of motifs (Additional file 1: Figure S6). None of these seed sequences are present at > 3% of the final population, and all motif frequencies were < 10%. Intriguingly, AptaTRACE did highlight that many of our more frequently identified motifs occur in paired regions as opposed to loop regions (Additional file 1: Figures S5 and S6, K-context traces). This finding suggests that methods analyzing pairing elements specifically may be more useful in understanding our data.

**Table 4:** Top AptaTRACE motifs

Motif	Seed *P*-Value	Seed Frequency(%)
ACTGCT	2.3e-4	6.55
ATACGG	2.5e-3	4.89
ACCAAG	4.1e-5	3.78
GGTATA	1.1e-3	2.54
AACGAA	4.6e-4	2.46

Due to our lack of non-binder data, we could not directly leverage all of the features in RNAcontext or RCK. To allow application of these tools to our data, we created a background data set (See Methods: Background set construction, BG _*Samp*_) to use as non-binder data (similar to approaches used in DeepBind [51]). We applied RCK to our binder and background data set, which identified motifs located in paired regions (Additional file 1: Figure S7). For k = 4 and k = 5, the motifs identified appear to be biased toward sequence that occurs in our non-constant region (see Methods, Additional file 1: Table S3). The k = 6 motif identified does not obviously share this bias. This motif occurs in 0.6% of the final data. Of note, this motif occurs in a paired region, further suggesting that examining paring elements more closely may be useful for this particular data set.

### Identification of structure motifs

The lack of enriched sequence motifs and global secondary structure conservation indicates the binding likely occurs in a substructure of the selected RNA sequences. The existence of a substructure is further supported by motifs identified by existing motif finders that appear to be in paired regions, and only account for a small fraction of the sequence pool. To identify potentially important substructures, we developed a novel approach that differs from existing methods by specifically focusing on stacking base-pairs. We represent stacked base-pairs as 2_2 nucleotide cyclic motifs (NCM) (See Methods: Identifying enriched/depleted secondary structure motifs) [52]. In this representation, each base-pair within a pairing region is part of two 2_2 NCMs, one with the base-pair above, and a second with the base-pair below. Therefore the sequence 5’-AGG-3’ base-paired to 5’-CCU-3’ would contain two NCMs: AU/GC and GC/GC. This representation is advantageous because NCMs discretize the secondary structure into smaller components and they have been used to great effect in improving RNA tertiary structure predictions [53].

NCM enrichment is calculated as the ratio of the mean NCM frequency for later rounds (9, 10, 11) relative to earlier rounds (4) or background (See Methods: Identifying enriched/depleted secondary structure motifs). As described for other methods above, 10^5^ sequences were repeatedly sampled from each round to determine NCM frequencies. Since this approach depends on structure predictions, we calculated NCM enrichment using both the minimum free energy (MFE) and the centroid structure, which better represents the ensemble of structures. Both structure representations have associated values for the free energy of the structure, which is inversely proportional to the thermodynamic stability (i.e. lower free energy structures have increased number of base-pairs). Thus, both representations capture trends such as increasing stability in later rounds. The NCM enrichment values derived from using the MFE structure and the centroid structure are moderately correlated (Additional file 1: Figure S8). Using the centroid structure reduces the NCM frequency, but the reduced frequency has small impact on enrichment. Therefore, we carried out the remaining enrichment analysis using the MFE structure.

**Fig. 4:**
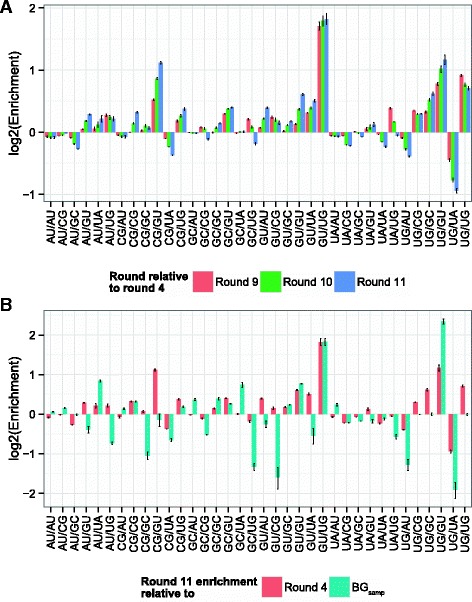
**a** Log2 fold change of NCMs averaged over 11 resamplings. The round 11 enrichment trends are consistent with the round 9 and round 10 enrichment. **b** Log2 fold change of NCMs averaged over 11 resamplings comparing the enrichment of round 11 vs. round 4 and round 11 vs. background created with sampled base frequency (BG _*samp*_). Error bars represent standard error

To identify significantly enriched NCMs, we also calculated the expected enrichment by comparing the NCM frequencies of the sampled sequences to background sequences, either created using uniform base frequencies (BG _*uni*_) or base frequencies based on our total sequence pool (BG _*samp*_) (See Methods: Background set construction). Our criteria for enrichment is that the NCM ratio of round 11 to round 4 must be significantly greater than the ratio of round 11 to background. Many NCMs are significantly enriched (AU/GU, AU/UG, CG/GC, CG/GU, GC/GU, GU/CG, UG/CG, UG/GC, UG/UG), while some are depleted (AU/CG, AU/GC, CG/AU, GC/AU) when compared against BG _*samp*_ (Fig. 4 b). There is significant overlap of enriched and depleted NCMs when comparing against BG _*uni*_ (Additional file 1: Figure S9). Interestingly, many of the enriched motifs contain a GU wobble pair, which could be a potential recapitulation of the natural binding site. Despite the lower percentage of round 4 sequences sampled, the enrichment analysis is robust to the sampling and identifies similar enriched, depleted, and unchanged NCMs relative to round 4 (Fig. 4 a). GU/UG and UG/GU appear to be highly enriched and have larger standard error. However, these NCMs are not significantly greater than background, and the high variability is due to low frequency, thus these are considered spuriously enriched NCMs.

The NCM enrichment in later rounds suggests selection for particular motifs. By treating clusters as “sequence families,” we used LASSO logistic regression to identify NCMs associated with cluster enrichment. Since the analysis depends the clustering, we re-clustered our sequence pool multiple times and found the clustering is relatively stable (Additional file 1: Figure S10). For each repeated clustering, we carried out LASSO regression and reduced our NCM predictors to those that appeared in majority of the models with *p*-value < 0.01. Using this method on both round 4 to round 11, and round 4 to round 10, we identified positive predictors CG/GU and GU/GC as well as negative predictors AU/GC and CG/UA that are found in both models (Table 5). CG/GU was identified by enrichment analysis as well, further indicating its importance.

**Table 5:** Representative NCMs that are significantly associated with cluster enrichment from first clustering

Rounds compared	NCM	Log odds (95% CI)	*P*-value
11 to 4	AU/GC	-3.39 (-4.95 - -1.85)	1.87e-5
	CG/GU	8.04 (3.07 - 13.16)	1.75e-3
	CG/UA	-5.53 (-7.75 - -3.37)	6.86e-7
	GU/GC	4.66 (2.08 - 7.27)	4.36e-4
10 to 4	AU/GC	-1.89 (-3.37 - -0.44)	0.0112
	CG/GC	3.11 (0.523 - 5.74)	0.0194
	CG/GU	9.45 (4.08 - 15.00)	6.78e-4
	CG/UA	-3.18 (-5.36 - -1.13)	2.47e-3
	GU/GC	4.51 (1.98 - 7.10)	5.61e-4
	UA/UA	-5.96 (-8.67 - -3.31)	1.31e-5

Given the overlap of predictors, we tested whether the logistic regression model for round 10 enrichment could predict future cluster enrichment (i.e. round 11 enrichment). Ideally, the same NCMs are selected throughout the SELEX process. After training on round 10 enrichment data, we tested the model by using cluster enrichment from each of the re-clustered data sets. However, this model offers a limited prediction accuracy (mean AUC=0.651), indicating some predictors are not readily identified (Additional file 1: Figure S11).

In order to ensure the 2_2 NCM was not part of a larger base-pair stack, we used Spearman correlation to identify any NCMs that often appear with each other. There is moderate correlation between some NCMs (*ρ*>0.6) (Additional file 1: Figure S12). However, this correlation is most likely spurious because repeated analysis with 3_3 NCMs does not show higher enrichment of these NCMs relative to BG _*samp*_ (Additional file 1: Figure S13).

### Experimental assessment of S15 binding affinity

In order to ensure our SELEX data provided an accurate reflection of binding sequences, we assayed a variety of sequences for binding affinity for S15 (Summarized in Table 6). We find many high frequency sequences had moderate affinity for S15 ranging from 19-85.6 nM (Table 6 A–F). Given the high diversity of the sequence pool, we also tested singleton sequences for binding, which revealed 6 of 8 singleton sequences also bind S15 (Table 6 G-N). Previous literature suggests that sequence enrichment is a better predictor of binding affinity [14]. We find that there is no correlation between the degree of enrichment and binding affinity (Additional file 1: Figure S14). Both depleted sequences tested bind S15 with moderate affinity (Table 6 O-P).

**Table 6:** Summary of experimentally tested sequences and their binding affinity

	Seq. Id	Cluster Id	K _d_ (nM)	Reason
A	98	52739	85	High freq.; High mean pairwise identity (> 90%)
B	101	6062	42	Most freq.; High mean pairwise identity (> 90%)
C	575	2903	62	High freq.; High mean pairwise identity (> 90%)
D	669	1792	25	High freq.; High mean pairwise identity (> 90%)
E	4778	851	19	High freq.; High mean pairwise identity (> 90%)
F	27773	517	2.8	High freq.; High mean pairwise identity (> 90%)
G	46474	63331	99	Singleton; Small cluster (≤100 seqs.)
H	355069	1307	123	Singleton; Small cluster (≤100 seqs.)
I	244064	4454	62	Singleton; Medium cluster (100<seqs.<1000)
J	158254	91212	31	Singleton; Singleton cluster (= 1 seq.)
K	279047	70316	77	Singleton; Singleton cluster (= 1 seq.)
L	4077	68	9.8	Singleton; Large cluster (≥1000 seqs.); Low mean pairwise identity cluster (< 90%)
M	170365	2293	Non-specific	Singleton; Low mean pairwise identity cluster (< 90%)
N	192209	3606	Non-specific	Singleton; Low mean pairwise identity cluster (< 90%)
O	4650	3969	38	Depleted; Medium cluster (100<seqs.<1000); low pairwise identity cluster (< 90%)
P	315173	5799	28	Depleted; Previously identified regulator [46]

We also tested sequences from clusters that are centered on high frequency sequences. When a sequence represents a large fraction of the cluster, we hypothesize that this sequence binds with high affinity while the remaining sequences “explore” the local sequence space. Fitting with our hypothesis, many high frequency sequences specifically bind S15 and are found in high mean pairwise identity cluster (Table 6 A-F). As a control, sequences from clusters with low mean pairwise identity not centered on high frequency sequences were also examined (Table 6 L-O). We find half of these sequences bind specifically, which suggests high identity clusters are more likely to contain S15 binders.

We use the enriched/depleted NCMs with our experimental data to build a model to identify potential binders (See Additional file 1: Methods for details). Due to the limited number of negative test cases, we use additional sequences from our background set to build a logistic regression model. The model suggests using enriched and depleted NCMs are good predictors of binding (mean AUC = 0.921) (Additional file 1: Figure S15).

## Discussion

The RNA binding sites of many proteins are complex in terms of both sequence and structure. In this work we sought to understand the pool of potential RNA-binding sites for *G. kaustophilus* ribosomal protein S15 using in vitro selection coupled with high-throughput sequencing (HT-SELEX). The high-throughput sequencing revealed a diverse population of sequences with over 95.3% of our sequences appearing only once in the population. We were able to cluster our data using a number of different methods. However, the large number of unique clusters produced did not share any obvious global structure or sequence characteristics. Existing strategies that have been applied to the analysis of other RBPs were unsuccessful at identifying any features that would explain a significant portion of our data. Many programs are not designed for the number or diversity of our sequence data. AptaTRACE and RCK, which take RNA structure into account, both return motifs that show a tendency toward regions involved in base-pairing rather than single stranded regions. However, the proportion of the data explained by any of these motifs is typically quite small.

HT-SELEX experiments often produce diverse sequence pools, and in many cases singleton sequences are regarded as “noise”. However, we examined several individual sequences from our population to show that both frequent sequences and those that only appear once in the population display specific binding with physiologically relevant dissociation constants (Table 6). Furthermore, no clear relationship between frequency, or degree of enrichment and dissociation constant was identified. This finding suggests that the diversity we observe may not be due to noise introduced by the selection process, but rather be a result of the large diversity of sequences with which *G. kaustophilus* ribosomal protein S15 may interact.

We developed a novel approach to analyzing HT-SELEX data for motifs that incorporate RNA structures. Our approach borrows from three-dimensional structure prediction [53], by considering all potential substructures or nucleotide cyclic motifs (NCMs) of a certain length. This approach is further necessitated by the complexity of the known RNA binding sites for S15 [39, 42, 43]. We repeatedly sampled sequences from each round to carry out our analysis. There are many enriched or depleted 2_2 NCMs relative to earlier rounds, with many of the enriched NCMs containing a GU wobble base-pair, which could be a potential recapitulation of the natural binding motif. By using LASSO regression, we effectively reduced the number of NCMs to potential predictors of enrichment.

Our algorithm is easily parallelized and the run time is increased proportionally to the number of secondary structure predictions. The run time falls on the shorter end of the spectrum compared to existing software, which can sometimes take a week to finish (Table 2). We have also demonstrated that the algorithm is robust to structure representation. Additionally, the NCM data is easily integrated into models to predict potential binders. Despite a limited number of validated binders and non-binders, the model accurately distinguish binders from background sequences. Surprisingly, our limited model classifies only 15.7% of the total sequence pool as potential S15 binders, suggesting many potential non-binders. Considering the proportion of binders found within our limited population of verified binder sequences, it appears that only a subpopulation of binding sequences can be identified using NCMs alone and that S15 likely can recognize additional features that are not captured by this data.

## Conclusion

Our analysis of the HT-SELEX data for the *G. kaustophilus* S15 suggests that this protein can bind a large diversity of sequences in vitro and our previous work demonstrated that half of the RNAs examined allowed regulation [46]. The analysis also suggests that the recognition motif is located in a combination of structure elements with little requirement on the sequence itself. This finding also illuminates a possible reason for the large sequence and structure diversity in natural S15 mRNA secondary structures. The approach we developed to analyze our data is broadly applicable to many other RBPs that have complex noncontiguous recognition motifs. By considering RNA secondary structure elements as building blocks (NCMs), we bring a novel approach to analyzing in vitro selection data for RNA-protein interactions that may primarily rely on specific local features in the context of a larger secondary structure.

## Methods

### High-throughput sequencing

We previously identified S15 binders using 11 rounds of SELEX [46]. We sequenced cDNA pools resulting from reverse transcription of the selected sequence pools after rounds 4, 9, 10, and 11. The sequence pools were sequenced using Illumina short read 100 nucleotide (nt) paired-end sequencing (Otogenetics Corporation). The expected length of the aptamer was 87 nt, composed of 30 nt PCR primers (bold), 30 nt variable region, and 27 nt non-constant (italicized) region to give a final form of 5’- **TGCGTAACGTACACT** -N30- *TCATTCTATATACTTTGGAGTTTTAAA* - **ATGTCTCTAAGTACT**. Sequences were filtered to have the correct primers, contain only standard bases, and match the forward strand (match the regular expression “TGCGTAACGTACACT[ATGC]+ ATGTCTCTAAGTACT”) with relative primer positioning such that the final sequence obtained was 79-100 nt. Sequences were also filtered such that every nucleotide’s PHRED quality score is ≥20. Any sequences shorter than 79 nt or containing duplicated T7 promoter sequence (5’-TAATACGACTCACTATA) were removed. These sequences are considered rapid amplifier sequences because they only contain T7, 5’, and 3’ sequences (See Additional file 1: Methods: Rapid amplifiers). The libraries are stored in separate FASTQ files for each round. The remaining sequences were stored in a MySQL database for speed and ease of access. For subsequent analysis, only the sequence contained between and including perfect primers was used. When calculating enrichment, the sequence counts were normalized to the total number of usable reads in that round.

### Clustering

#### Sequence

In order to determine a cluster threshold, sequences from rounds 10 and 11 with >100 total counts were used as initial cluster centroids to compare to the remaining sequences. The normalized edit distance (normalized Levenstein distance) was calculated as the $\frac {\text {edit distance(s1 and s2)}}{\text {max length(s1 and s2)}}$. As a computational optimization, the regions of the aptamer corresponding to the primers (5’- TGCGTAACGTACACT and 5’-ATGTCTCTAAGTACT) were removed for the purposes of sequence comparison as these sequences are identical in all of our filtered sequences. CD-HIT-est [47] was used for nucleotide clustering with the following options: compare positive strand only (–r 0), mismatch penalty –1, gap penalty –1, gap extension 0 and cluster threshold of 85% (-c 0.85). The mismatch penalty and gap open penalty are both the same value to minimize the effect of single base variation or deletions in the variable region. The gap extension is set to 0 because it heavily penalized short stretches of base differences in the variable region thus creating many more singleton clusters. The output from CD-HIT was imported into a MySQL database for speed and ease of access.

#### Structure

RNAclust.pl + LocARNA will cluster sequences based on sequence and structure. We used the default parameters, 8 CPU threads and “–sparse” for the LocARNA option. For these comparisons, the full expected aptamer sequence was utilized.

#### Intra/inter-cluster ensemble distance

As an alternative to clustering sequences based on sequence identity, we cluster sequences using their secondary structures by comparing structures using ensemble distance. For these comparisons, the entire expected aptamer sequence including primer sequences was utilized. We find that similar sequences tend to fold into similar structures. Therefore, as a run time optimization, we focus on frequent sequences within existing CD-HIT clusters, which allows us to estimate a cluster structure and reduces the number of sequences that are folded. The clusters used for analysis were selected from the CD-HIT clusters using the following criteria: > 100 sequences and > 90% mean identity to the CD-HIT cluster seed. Secondary structure prediction was done using the Vienna RNAfold package [54]. The ensemble distance was calculated by first predicting the secondary structure ensemble using ‘RNAfold -p’. The ensemble distance is the mean base-pair distance between all possible structures of two input sequences [55]:

1 $$ \frac{1}{|A|}\sum_{(i,j) \in A \cup B}{\left(P_{ij}^{A} - P_{ij}^{B}\right)^{2}}  $$

where *i*<*j* and *P*_*ij*_ is the probability of a nucleotide at position *i* paired to a nucleotide at position *j* and |*A*| is the length of structure A. Structures A and B must be the same length.

Intra-cluster distance was calculated by taking 1000 (or fewer) distinct sequences from each of the clusters meeting our criteria. Then ensemble distance was calculated in a pairwise fashion.

Inter-cluster distance was calculated using the top 100 most frequent sequences from each cluster. Structures in each cluster were compared in a pairwise manner to structures in the other cluster.

### Sequence and structure motif identification

We applied a variety of existing motif finder programs to our sequence pool: DREME, GLAM2, AptaTRACE, and RNAcontext/RCK. For all programs, we used the same sample, which is created by sampling 10^5^ sequences from each round of selection for a total of 4∗10^5^ sequences, unless otherwise noted.

#### Sequence

The parameters for DREME were motifs of length k such that 3≤*k*≤8, no reverse complement, and stop after the top 10 motifs are identified. GLAM2 parameters: motifs of length k such that 3≤*k*≤8, and 50000 iterations.

#### Sequence and structure

AptaTRACE was run with default parameters (k-mer length 6, singleton threshold 3), designating the 5’ (TGCGTAACGTACACT) and 3’ (TCATTCTATATACTTTGGAGTTTTAAAATGTCTCTAAGTACT) primer and constant regions, with SFold [56] as the RNA folding program. We have chosen to run RCK on our data because it is an newer extension of RNAcontext. RCK was run with motif length k such that 4≤*k*≤8. RCK additionally requires intensities for bound and unbound sequences as part of its training and test set data. As input, the sample sequences were considered bound and had intensity equal to 1. Sequences created from sampled base distribution (BG _*samp*_) (See Methods: Background set construction) were used as unbound sequences and had intensity equal to –1. For all other parameters, we used the default values.

### Background set construction

The background sequence set variable region was created using either a uniform (BG _*uni*_) or a sampled base distribution (BG _*samp*_). The sampled base frequency is determined using the variable regions from the sequence pool. The variable region was identified by minimizing the Levenshtein distance between our known non-constant region sequence (TCATTCTATATACTTTGGAGTTTTAAA) and a sliding window of length 20 along the given input sequence.

Any mutations to the non-constant region was simulated using the “mutation rate” derived from the non-constant region of round 11 sequences. The mutations were categorized as point mutation, insertion, or deletion. The sequence was simulated by choosing the site(s), which is governed by the Poisson distribution, and type(s) of mutation based on the overall mutation frequency. Then the resulting mutation is selected based on the observed mutational frequency. The final simulated sequence was generated by concatenating the primers, a simulated variable region (30 bases chosen with uniform or observed probability) and a simulated non-constant region in the proper order.

### Identifying enriched/depleted secondary structure motifs

The structural motifs we identify are derived from the 2_2 and 3_3 nucleotide cyclic motifs (NCM) [53]. We modified the naming convention to be more base-pair centric — N1_N2 <sequence> such that the N1 and N2 designate the length of the 5’ and 3’ strands, respectively. The <sequence> represents the order of stacking base-pairs starting at the 5’ end.

To calculate NCM enrichment, NCMs are counted by sampling 10^5^ distinct sequences corresponding to the entire expected aptamer sequence without replacement from each round. For each sequence, the MFE or centroid structure is predicted using Vienna RNAfold [54] and each possible 2_2 or 3_3 NCM stack is counted. Similar to calculating k-mer frequency, NCM frequency is calculated by normalizing the NCM count to the total number of NCMs per sequence and number of sequences sampled. NCM enrichment/depletion is calculated by the ratio of the mean NCM frequency between any two rounds. The code for calculating NCM enrichment is located at https://github.com/ship561/hts-exploration.

In order to identify enriched NCMs, we repeatedly calculate NCM enrichment relative to both round 4 and a background set. The NCM enrichment relative to background provides an “expected” baseline enrichment value. NCMs are considered significantly enriched when the average NCM enrichment relative to round 4 is higher than average expected NCM enrichment (*p*-value < 0.001). Significance is calculated using the Wilcoxon rank sum test [57].

### LASSO Logistic regression models

Logistic regressions and LASSO were done in the R project [57]. Only clusters with > 100 sequences were used, as these clusters are likely to contain sequences from different rounds. Additionally, only clusters containing sequences from both earlier and later rounds, and with a sequence frequency ratio from later to earlier rounds exceeding a minimum threshold. Due to the variation in sequences per round, this minimal threshold varies depending on which rounds are compared. Cluster enrichment is defined as a cluster that contains a higher frequency of sequences from a later round (10 or 11) than an earlier round (4 or 9). For two rounds X and Y (where X>Y), cluster enrichment is calculated using

2 $$ \text{Cluster enrichment} = \frac{ \frac{\text{\# total sequences in cluster of round X}} {\text{\# total sequences in round X}}} {\frac{\text{\# total sequences in cluster of round Y}} {\text{\# total sequences in round Y}}}  $$

For example, a minimal threshold for cluster enrichment between rounds 11 and 4 is calculated by considering a cluster composed of two sequences — a single sequence from round 11 and another sequence from round 4. Thus, for round 11 (r11) sequences to be considered enriched, the ratios r11:r4 > 7.61 or r11:r9 > 0.7468. For round 10 (r10) sequences to be considered enriched, the ratios r10:r4 > 8.61 or r10:r9 > 0.8451. For the training set, a 1:1 ratio of enriched vs depleted clusters were used. The number of enriched and depleted clusters for each re-clustering is summarized in Additional file 1: Table S4.

We re-clustered the sequences multiple times using CD-HIT because it employs a greedy clustering algorithm that is sensitive to the starting sequence order. For each CD-HIT re-clustering, NCM predictors are selected automatically by LASSO logistic regression. Predictors are retained if they appear in 3 out of 5 re-clusters with a significant *p*-value < 0.01.

### RNA/Protein preparation

The aptamer sequence was synthesized using assembly PCR from overlapping oligos (from IDT) with the T7-promoter sequence added within the forward primer sequence. T7 RNA polymerase [58] was used to transcribe RNA and transcription reactions were purified by 6% denaturing PAGE. Bands were visualized using UV shadow, excised, and the RNA eluted (in 200 mM NaCl, 1 mM EDTA ph 8, 10 mM Tris-HCl pH 7.5) and ethanol precipitated. Purified RNA (10 pmol) was 5’-labeled with ^32^P-ATP and purified as previously described [59]. Protein expression and purification was conducted as described previously [40].

### Filter binding assay

As done in Slinger et. al 2015, [46] a fixed amount of 5’^32^P-labeled RNA (1000 cpm, <1 nM) was renatured for 15 minutes at 42°C, then incubated with serial dilution of *G. kaustophilus* S15 in Buffer A (50 mM-Tris/Acetate, pH 7.5, 20 mM Mg-acetate, 270 mM KCl, 5 mM dithiothreitol, 0.02% bovine serum albumin) for 30 minutes at 25°C. Nitrocellulose membrane (GE Healthcare) was used to collect RNA-S15 complexes and positively charged nylon membrane (GE Healthcare) was used to collect unbound RNA under suction in a filter binding apparatus. Membranes were air-dried 5 minutes and the fraction bound quantified by imaging membranes on a phosphorimager screen. Radioactivity counts per sample on each membrane were measured using GE Healthcare STORM 820 phosphorimager and ImageQuant. For each sample the fraction bound (Fb) corresponds to

3 $$ Fb = \frac{\text{counts nitrocellulose}}{\text{counts nitrocellulose} + \text{counts nylon}}  $$

Since Fb is known, to determine the K _d_ and the Hill coefficient (*n*), the resulting values were fit to the equation:

4 $$ Fb = Min\%+ \frac{Max\%-Min\%}{1+\left(\frac{K_{d}}{[S15]}\right)^{n}}  $$

where [S15] corresponds to the concentration of S15 in the reaction and *Min* *%* and *Max* *%* correspond to the minimum and maximum fraction bound, respectively. The residuals were minimized using the nonlinear least squares estimate (nls) in R to find both the Hill coefficient (*n*) and the K _d_.

## Additional file

Additional file 1 Supplemental data. The file is in a PDF format. It contains methods for identifying rapid amplifying sequences and calculating “belief” in our inter-cluster distance comparisons. It contains additional **Tables S1**–**S4** showing the percentage of rapid amplifier sequences per SELEX round (**Table S1**), the low inter-cluster distance clusters with their calculated belief (**Table S2**), the top RCK motif frequencies categorized by region (**Table S3**), and the results of the LASSO regression obtained after re-clustering the data (**Table S4**). Additional Figures (**S1**–**S15**) showing: pairwise distance between all high frequency sequences (**Figure S1**), distribution of intra-cluster structure distance (**Figure S2**), distribution of belief to support inter-cluster ensemble distance (**Figure S3**), a representative motif from GLAM2 (**Figure S4**), representative results from AptaTrace running on a 33% sampling of the data (**Figure S5**), results from AptaTrace running on the entire dataset (unequal number of sequences in each round) (**Figure S6**), motifs and contexts identified by RCK (**Figure S7**), the correlation between using the MFE and centroid structures to calculate NCM enrichment (**Figure S8**), NCM enrichment relative to round 4 and BG _*uni*_ (**Figure S9**), CD-HIT cluster stability (**Figure S10**), the model performance for classification of enriched clusters (**Figure S11**), correlation between 2_2 NCMs, and 3_3 NCM enrichment relative to background sequences generated using either uniform or sampled base probabilities (**Figures S12** and **S13**), the relationship between k _d_ and enrichment (**Figure S14**), and the model performance classifying sequences as “binders” or “non-binders” using enriched/depleted NCMs as features (**Figure S15**). (PDF 1270 kb)

## Abbreviations

AUC: Area under the curve
BG _*uni*_: Background set (Uniform base probability)
BG_*samp*_: Background set (Sampled base probability)
DBP: DNA-binding protein
Fb: Fraction bound
HT-SELEX: High-throughput sequencing SELEX
HTS: High-throughput sequencing
MFE: Minimum free energy
NCM: Nucleotide cyclic motif
nt: nucleotide
RBP: RNA-binding protein
SELEX: Systematic evolution of ligands by exponential enrichment
