# Supplementary material for: Recognizing RNA structural motifs in HT-SELEX data for ribosomal protein S15
Source: BMC Bioinformatics. 2017 Jun 6;18:298. doi: 10.1186/s12859-017-1704-y (PMC5461778; doi:10.1186/s12859-017-1704-y)
Supplement: Additional file 1 — Supplemental data. The file is in a PDF format. It contains methods for identifying rapid amplifying sequences and calculating “belief” in our inter-cluster distance comparisons. It contains additional Tables S1–S4 showing the percentage of rapid amplifier sequences per SELEX round (Table S1), the low inter-cluster distance clusters with their calculated belief (Table S2), the top RCK motif frequencies categorized by region (Table S3), and the results of the LASSO regression obtained after re-clustering the data (Table S4). Additional Figures (S1–S15) showing: pairwise distance between all high frequency sequences (Figure S1), distribution of intra-cluster structure distance (Figure S2), distribution of belief to support inter-cluster ensemble distance (Figure S3), a representative motif from GLAM2 (Figure S4), representative results from AptaTrace running on a 33% sampling of the data (Figure S5), results from AptaTrace running on the entire dataset (unequal number of sequences in each round) (Figure S6), motifs and contexts identified by RCK (Figure S7), the correlation between using the MFE and centroid structures to calculate NCM enrichment (Figure S8), NCM enrichment relative to round 4 and BG uni (Figure S9), CD-HIT cluster stability (Figure S10), the model performance for classification of enriched clusters (Figure S11), correlation between 2_2 NCMs, and 3_3 NCM enrichment relative to background sequences generated using either uniform or sampled base probabilities (Figures S12 and S13), the relationship between k d and enrichment (Figure S14), and the model performance classifying sequences as “binders” or “non-binders” using enriched/depleted NCMs as features (Figure S15). (PDF 1270 kb) [file 12859_2017_1704_MOESM1_ESM.pdf]

Supplemental methods, tables, and figures for Pei *et. al.*

1 Methods

Rapid amplifier

We remove short sequences of 79 nt that are only composed of the T7 promoter, 5' primer, and 3' primer. These sequences do not contain a variable region and appear to become an increasingly large fraction ($>$ 20%) of the sequence population after the PCR amplification step (Table S1). Experimental results show these sequences do not specifically bind to the *G. kaustophilus* S15; therefore, we remove them from our sequence pool during analysis.

Calculating belief for structure distances

Only structures of the same length are comparable using ensemble distance leading to some of these distances being calculated using a small number of structures. To identify cluster pairs impacted by this artifact, we calculated the ratio of actual comparisons versus the number of possible comparisons (Supp. Figure S4). If the ratio was less than 0.01, the ensemble distance was ignored because it was likely calculated using only one sequence per cluster. This additional screen removed all clusters that appear to be similar.

$$belief = \log \frac{count}{min(10000, (cid1size * cid2size))} \tag{1}$$

where $count$ is the number of pairwise comparisons between 2 clusters (cid1, cid2) and $cid1size$ is the size of cluster1 and $cid2size$ is the size of cluster2.

Classifying S15 binders using the NCM model

When predicting potential binders, we use our experimentally validated sequences as positives (14 positive, 2 negatives) and 16 sampled sequences from BG_{samp} as negatives. For each sequence, we calculated the NCM frequency. The model uses the established enriched/depleted NCMs as predictors (AU/GU, AU/UG, CG/GC, CG/GU, GU/AU, GU/CG, GU/UA, UG/CG, UG/GC, GC/GC, GC/UA). Some NCMs are removed because of singularities. Since our data set is small, we re-sampled background sequences and average the performance over multiple samples.

2 Tables

Table S1: Percentage of rapid amplifier sequences in the SELEX sequence data separated by round.

Round	Percent rapid amplifier (%)
4	1.1
9	73.6
10	23.2
11	30.9

Table S2: Clusters that have a mean structure distance less than the median intra-cluster distance of 0.0946 were also considered structurally similar.

Cluster1	Cluster1 size	Cluster2	Cluster2 size	Mean ensemble distance	belief
3543	253	21035	294	0.09324413	-1.744
5300	134	3543	253	0.09345742	-2.008
5300		72036	402	0.06441246	-2.698
5300		82519	911	0.05355445	-3.339
12222	209	82519	911	0.08549975	-2.031
72036	402	1290	601	0.08523696	-2.136
72036		21035	294	0.08567548	-1.920
82519	911	1290	601	0.08320683	-2.958
82519		2293	390	0.09279544	-2.823
82519		21035	294	0.08129743	-2.040

Table S3: Top RCK motif frequency

Motif	Var	Var+non-const.
TTTT	0.018	0.774
TTAAA	0.019	0.845
AAATGT	0.006	0.007
AAATGTC	1.4E-3	2.9E-3
AAATGTCT	3.3E-4	4.8E-4

Table S4: Number of enriched clusters from each clustering run.

Cluster run	Depleted	Enriched	Total
1	1140	2079	3219
2	1151	2073	3224
3	1140	2083	3223
4	1152	2051	3203
5	1162	2062	3224

3 Figures

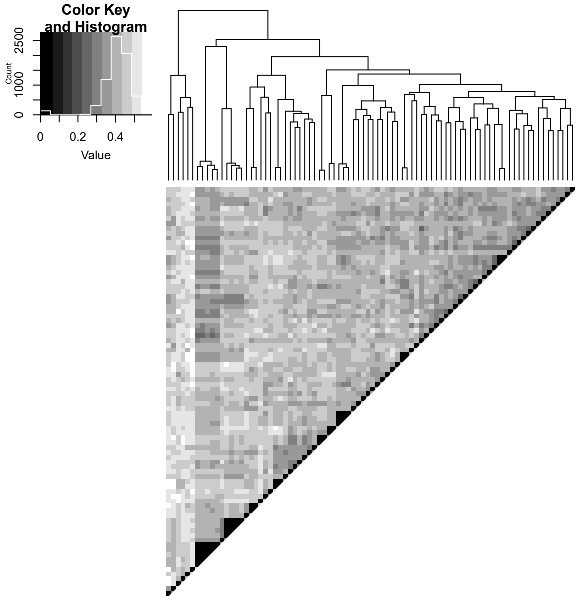

Figure S1: Heatmap of pairwise edit distance between the 84 multitons reveals very few multiton sequences can be grouped together (black). Majority of multitons are unrelated (> 10%) to any other multiton sequence. Values are symmetrical across the diagonal.

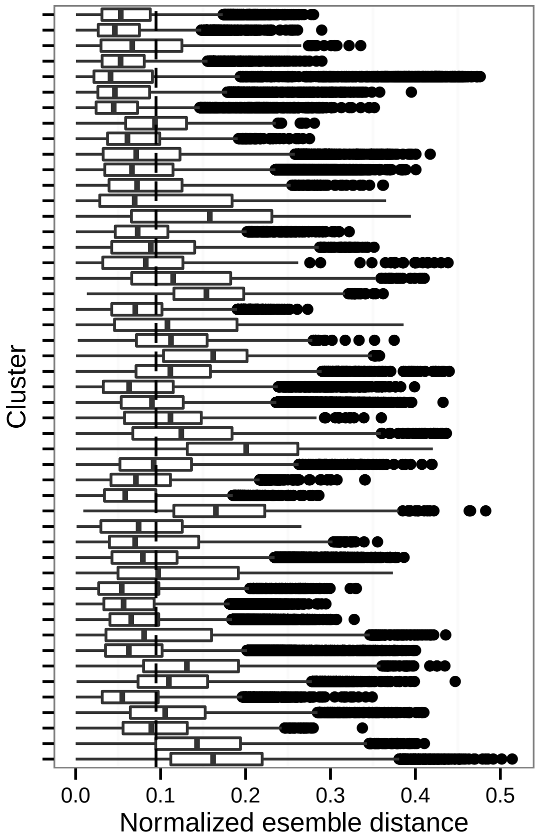

Figure S2: Distribution of intra-cluster ensemble distances by cluster. Box edges represent the first and third quartiles with the middle being the median. Clusters with the following criteria were selected: >100 sequences, and >90% mean identity to the seed. The line represents the median intra-cluster distance at 0.0898. The mean distance was 0.0946.

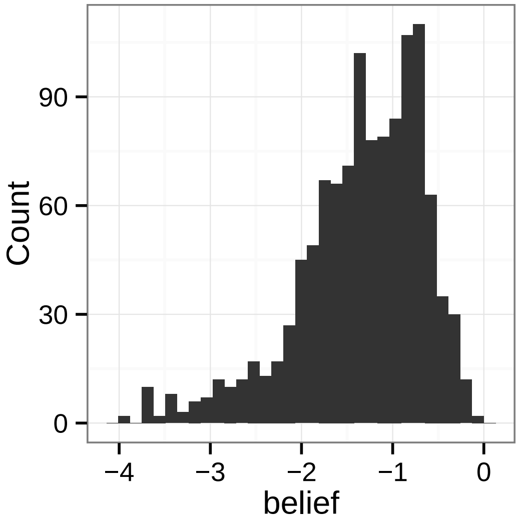

Figure S3: Distribution of belief (see supplemental methods for definition) in the evidence supporting our inter-cluster ensemble distance. The belief shows how much we believe in the evidence. Because certain clusters do not have enough pairwise comparisons, the average distance is somewhat biased to be low. The threshold for believable was set at the beginning of the left-tail, such that belief ≥ -2.

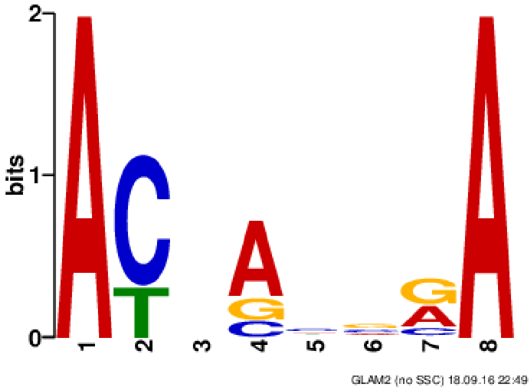

Figure S4: A logo representing the top motif from running GLAM2 over our sampled data set. This motif is unlikely to be the S15 recognition motif because of the low conservation of majority of the motif other than the two A's. Additionally, this motif is not significant (E-value=1).

ID	Motif Profile	Seed	Seed P-value	Seed Freq.	Motif Freq.	K-context Trace
1)	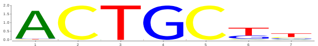	ACTGCT	2.302E-4	6.55%	8.28%	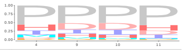
2)	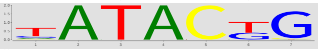	ATACGG	2.543E-3	4.89%	6.53%	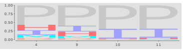
3)	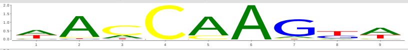	ACCAAG	4.101E-5	3.78%	8.93%	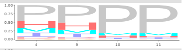
4)	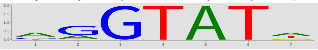	GGTATA	1.088E-3	2.54%	5.48%	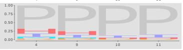
5)	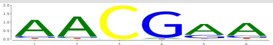	AACGAA	4.6E-4	2.46%	17.53%	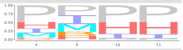
6)	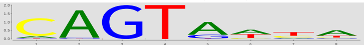	CAGTAA	8.714E-4	2.25%	7.92%	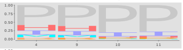
7)	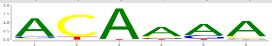	ACAAAA	2.608E-5	2.23%	16.43%	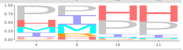
8)	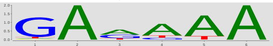	GAAAAA	3.465E-6	2.11%	10.80%	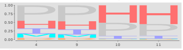
9)	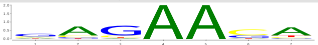	AGAACA	1.074E-3	2.05%	16.50%	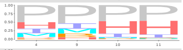
10)	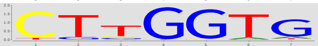	CTTGGT	1.051E-4	2.02%	11.81%	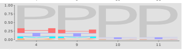
11)	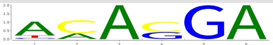	ACACGA	1.149E-3	1.76%	4.62%	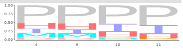

Figure S5: Results generated by AptaTRACE running on a 33% sampling of our HTS-SELEX data. The top motif is similar to a motif generated by DREME (See Table 3 in main text). The frequency of the seed sequences identified is relatively low (<10%), and even partially degenerate motifs are typically present at $<$ 15% of the sequences. Of note, most the motifs identified appear in paired regions (designated by P in the K-context traces).

ID	Motif Profile	Seed	Seed P-value	Seed Freq.	Motif Freq.	K-context Trace
1)	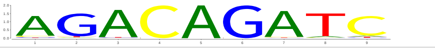	GACAGA	1.008E-5	2.83%	6.65%	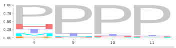
2)	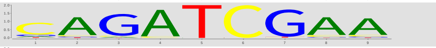	AGATCG	2.34E-5	2.69%	9.02%	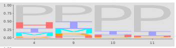
3)	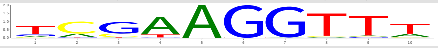	GAAGGT	3.148E-5	2.31%	6.49%	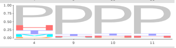
4)	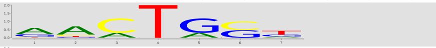	ACTGCT	1.13E-6	2.26%	4.26%	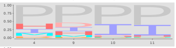
5)	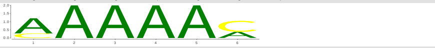	AAAAAC	3.991E-6	2.11%	4.49%	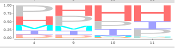
6)	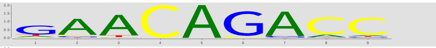	AACAGA	4.789E-6	2.07%	5.15%	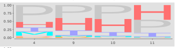
7)	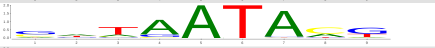	TAATAC	4.973E-6	2.05%	6.71%	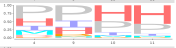
8)	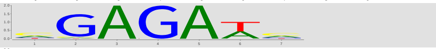	GAGAAC	1.58E-5	1.90%	7.07%	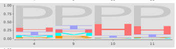
9)	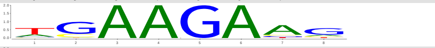	GAAGAA	2.502E-6	1.76%	4.47%	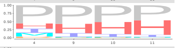
10)	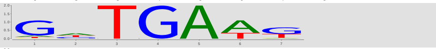	GATGAA	6.601E-6	1.76%	3.92%	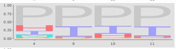
11)	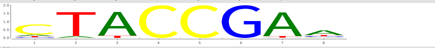	TACCGA	1.368E-5	1.55%	2.41%	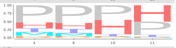
12)	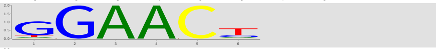	AGGAAC	5.309E-5	1.43%	3.02%	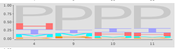
13)	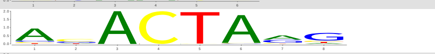	ACACTA	3.705E-6	1.31%	7.97%	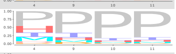
14)	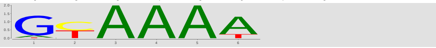	GCAAAA	3.823E-5	1.19%	2.59%	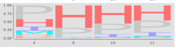
15)	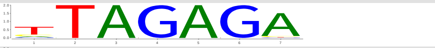	TAGAGA	2.009E-8	1.11%	1.32%	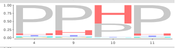
16)	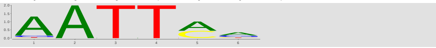	GAATTA	6.419E-6	1.05%	2.10%	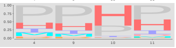

Figure S6: Results generated by AptaTRACE running the entire HTS-SELEX data. The frequency of the seed sequences identified is uniformly very low (<4%), and even partially degenerate motifs are typically present at <10% of the sequences. Of note, several of the high-scoring motifs appear in paired regions (designated by P in the K-context traces).

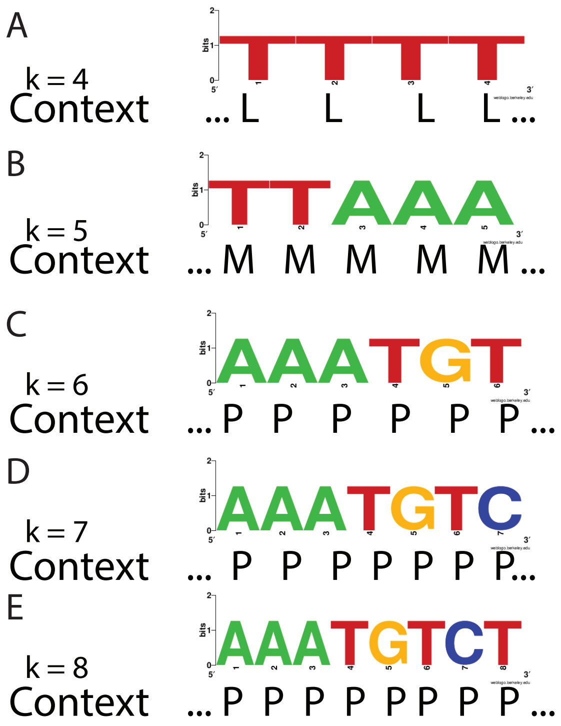

Figure S7: Motifs and context identified by RCK. The context alphabet is abbreviated as such: loops (L), miscellaneous (M) - includes multiloops and bulges, and paired (P). A) Motif width of length 4 is preferentially found in loops with a motif frequency of 1.8% in the variable region alone and 77.4% in the combined variable + non-constant region. B) Motif width of length 5 shows a preferential context in multiloops and buldges. This motif frequency is 1.9% in the variable region alone and 84.5% in the variable + non-constant region. C) Motif width of length 6 is also preferentially found in base-paired regions. The motif frequency in our data set is 0.6% in the variable region and 0.7% in the variable + non-constant region.

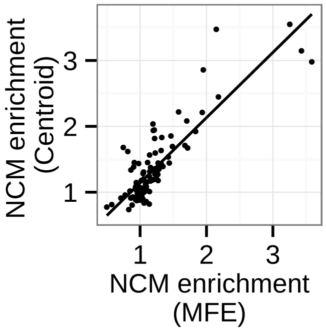

Figure S8: NCM enrichment as calculated using the minimum free energy (MFE) or the centroid structure. The NCM enrichment of later rounds (9, 10, 11) relative to round 4 shows moderate correlation (r^2=0.771) between the structure representations.

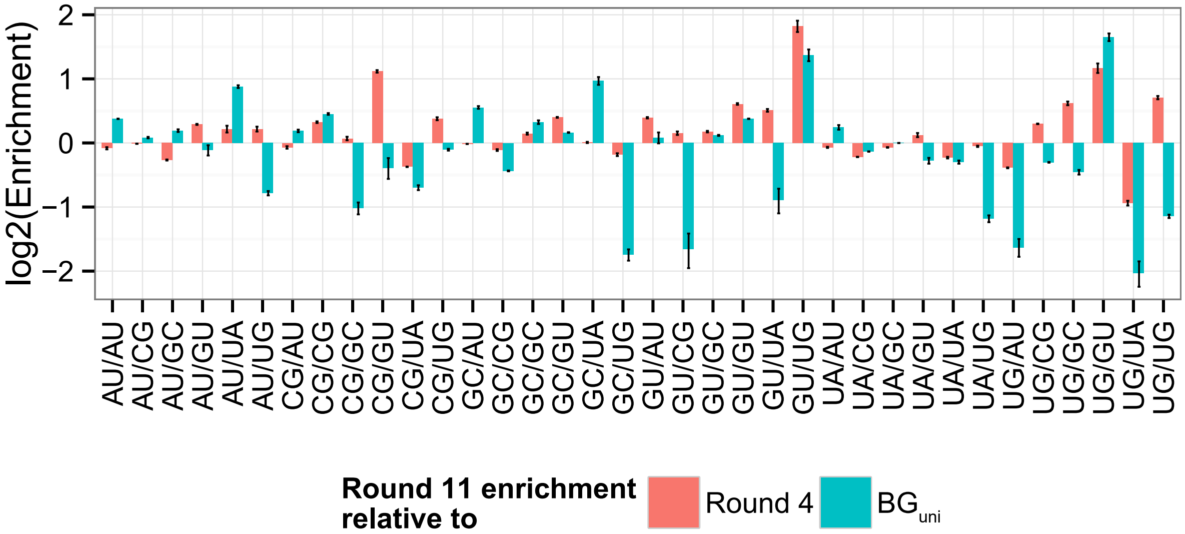

Figure S9: Log2 fold change of NCMs averaged over 11 re-samplings comparing the enrichment of round 11 vs. round 4 and round 11 vs. BG_{uni}. Error bars represent standard error.

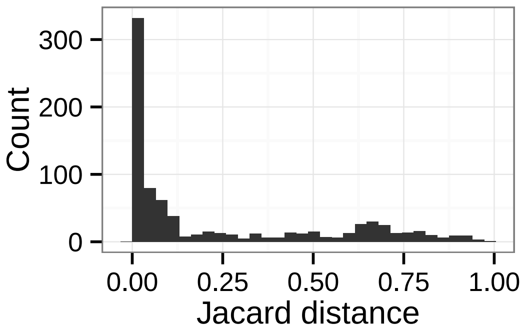

Figure S10: The CD-HIT clusters for high frequency sequences are relatively stable and many sequences often appear together despite multiple clustering runs. We show the distribution of the average the Jacard distance over 5 clustering runs. Occasionally, sequences are put into a different cluster, represented by high Jacard distance, but it is rare compared to being put into a similar cluster.

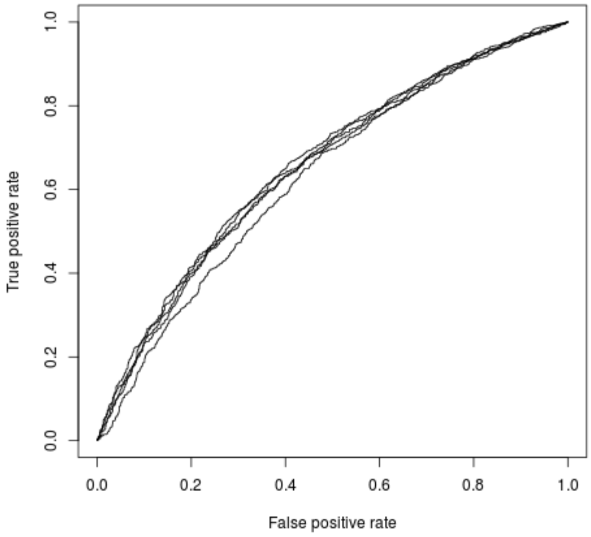

Figure S11: Receiver operator characteristic (ROC) curves showing the model performance on classifying clusters as enriched for later round sequences. Each line represents the LASSO logistic regression model applied to a separate CD-HIT re-clustering run. The model shows similar classification performance across all runs. The mean area under the curve (AUC) is 0.651.

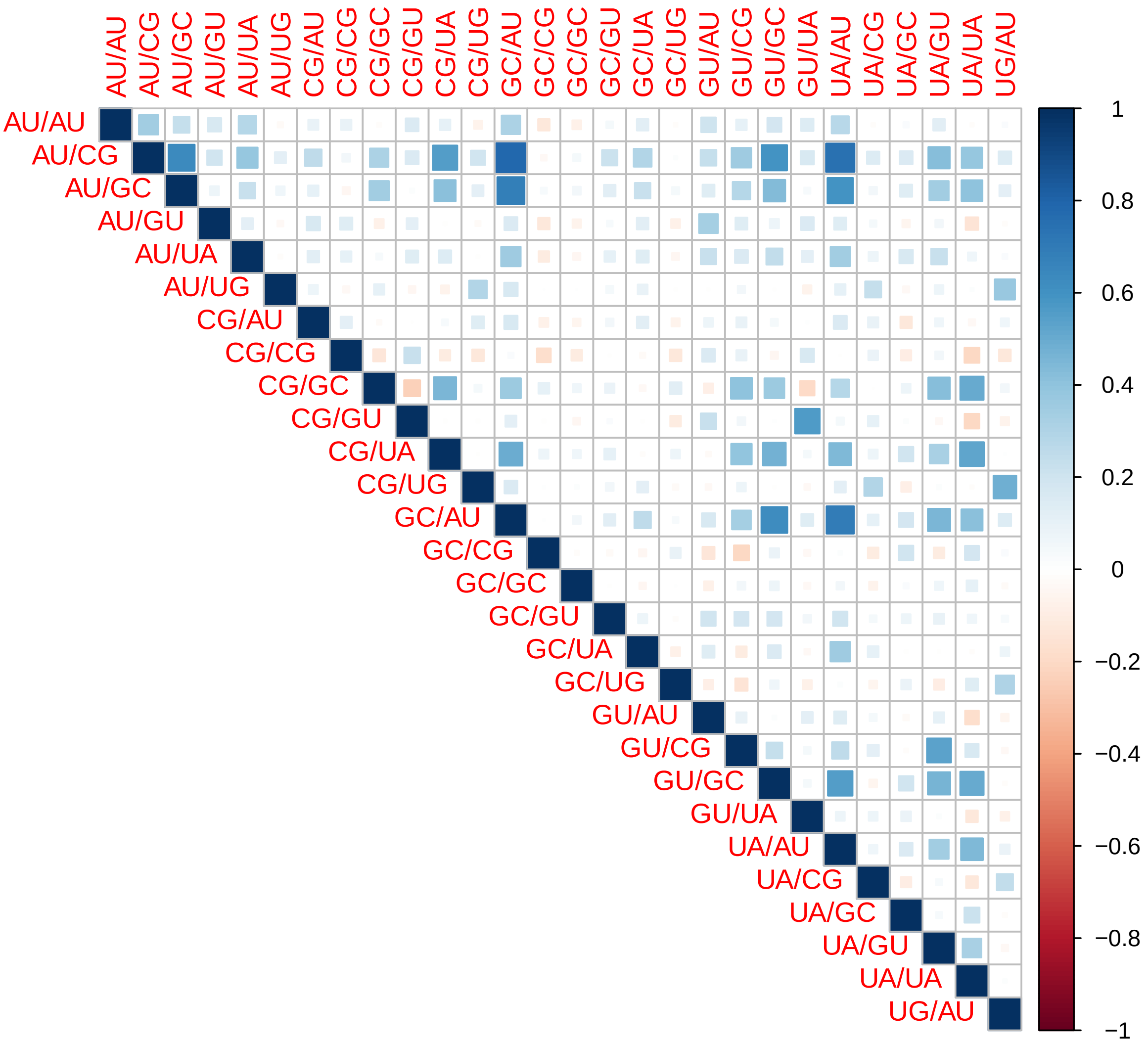

Figure S12: Spearman correlation matrix between all 2_2 NCMs with greater than 10k counts per round. The matrix is symmetrical around the diagonal. The larger and darker squares indicate stronger correlation. Positive correlation indicates the NCM pair could be combined as part of a larger binding motif. The correlation between GTTA and CGGT is 0.647. The correlation between TATA and CGTA is 0.522.

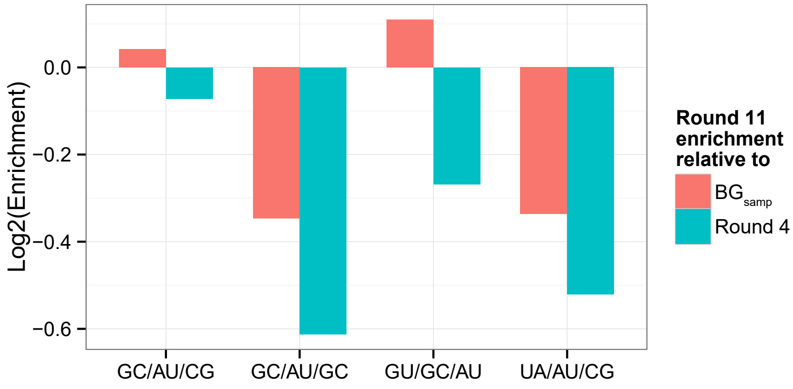

Figure S13: The enrichment/depletion of 3_3 NCMs as compared to round 4 or BG_{samp}. These NCMs are composed of 2_2 NCMs that often appear together suggesting a potentially larger motif. The ratio suggests these larger motifs are more depleted in later rounds than expected.

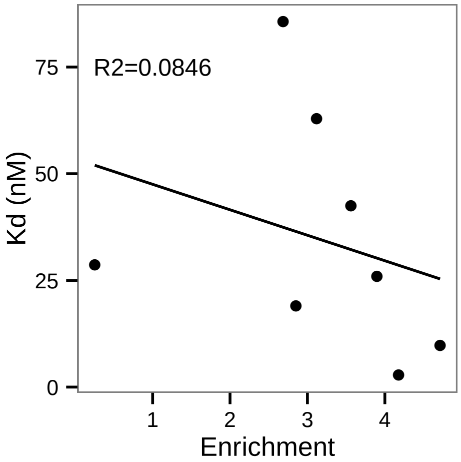

Figure S14: Linear regression showed that the sequence enrichment did not predict the K_d. The regression line has an R^2 value of 0.0846.

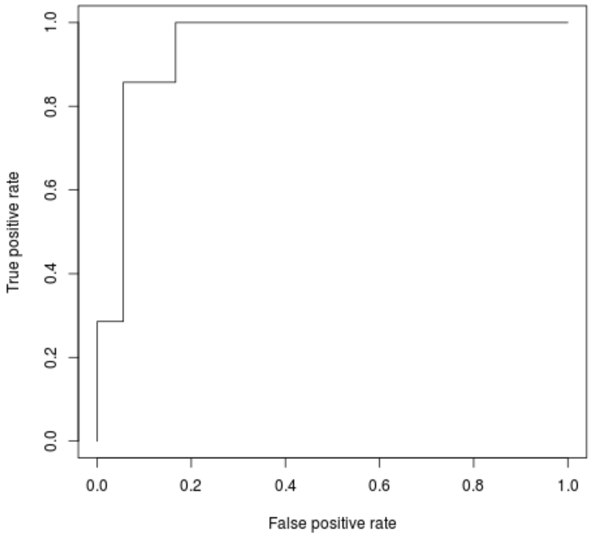

Figure S15: The logistic regression model using significantly enriched/depleted NCMs as predictors is applied to multiple re-sampled data sets. Each data set is composed of the experimentally tested sequences and non-binder sequences from BG_{samp}. A representative Receiver operator characteristic (ROC) curve shows the model performance on classifying sequences as either "binders" or "non-binders". After training our model on multiple data sets, the mean area under the curve (AUC) is 0.921.
